# Defective binding of SPINK1 variants is an uncommon mechanism for impaired trypsin inhibition in chronic pancreatitis

**DOI:** 10.1016/j.jbc.2021.100343

**Published:** 2021-01-28

**Authors:** András Szabó, Vanda Toldi, Lívia Diána Gazda, Alexandra Demcsák, József Tőzsér, Miklós Sahin-Tóth

**Affiliations:** 1 Department of Biochemistry and Molecular Biology, Faculty of Medicine, University of Debrecen, Debrecen, Hungary; 2 Center for Exocrine Disorders, Department of Molecular and Cell Biology, Boston University, Henry M. Goldman School of Dental Medicine, Boston, Massachusetts, USA; 3 Doctoral School of Molecular, Cell and Immune Biology, University of Debrecen, Debrecen, Hungary; 4 Department of Surgery, University of California Los Angeles, Los Angeles, California, USA

**Keywords:** trypsin inhibitor, pancreatitis, tyrosine sulfation, reactive-site peptide bond, equilibrium binding assay, HEK 293T, human embryonic kidney 293T cell, HRP, horseradish peroxidase, Hu1, human cationic trypsinogen, Hu2, human anionic trypsinogen, SPINK1, serine protease inhibitor Kazal type 1

## Abstract

The serine protease inhibitor Kazal type 1 (SPINK1) protects the pancreas from intrapancreatic trypsin activation that can lead to pancreatitis. Loss-of-function genetic variants of SPINK1 increase the risk for chronic pancreatitis, often by diminishing inhibitor expression or secretion. Variants that are secreted normally have been presumed to be pathogenic because of defective trypsin inhibition, but evidence has been lacking. Here, we report quantitative studies on the inhibition of human trypsins by wildtype SPINK1 and seven secreted missense variants. We found that tyrosine sulfation of human trypsins weakens binding of SPINK1 because of altered interactions with Tyr43 in the SPINK1 reactive loop. Using authentic sulfated human trypsins, we provide conclusive evidence that SPINK1 variants N34S, N37S, R65Q, and Q68R have unimpaired inhibitory activity, whereas variant P55S exhibits a small and clinically insignificant binding defect. In contrast, rare variants K41N and I42M that affect the reactive-site peptide bond of SPINK1 decrease inhibitor binding by 20,000- to 30,000-fold and three- to sevenfold, respectively. Taken together, the observations indicate that defective trypsin inhibition by SPINK1 variants is an uncommon mechanism in chronic pancreatitis. The results also strengthen the notion that a decline in inhibitor levels explains pancreatitis risk associated with the large majority of SPINK1 variants.

The pancreas secretes a variety of digestive enzymes, including several isoforms of the proteases trypsin, chymotrypsin, elastase, and carboxypeptidase. Trypsin isoforms, encoded by the serine protease 1, 2, and 3 genes, are commonly denoted as cationic trypsin, anionic trypsin, and mesotrypsin, based on their relative isoelectric points. To protect the pancreas, digestive proteases are secreted as inactive precursors, named zymogens, which become activated in the duodenum. The intestinal transmembrane protease enteropeptidase activates trypsinogen to trypsin, which, in turn, activates chymotrypsinogens, proelastases, and procarboxypeptidases to their active form (1). Among the digestive proenzymes, trypsinogen has the unique ability to undergo autoactivation, a self-amplifying bimolecular reaction in which trypsin activates trypsinogen. If this occurs inside the pancreas, the inflammatory disorder pancreatitis develops (2). As a defense mechanism against trypsinogen autoactivation and unwanted intrapancreatic trypsin activity, the pancreas secretes the serine protease inhibitor Kazal type 1 (SPINK1), also known as the pancreatic secretory trypsin inhibitor. This 6.2-kDa protein constitutes about 0.1 to 0.8% of the total protein in human pancreatic juice, which, in molar terms, corresponds to 2 to 13% of the trypsinogen content ([2] and references therein).

In 2000, Witt *et al*. (3) and later Pfützer *et al*. (4) reported that the N34S variant of SPINK1, which is present in the general population with around 1% frequency, is a strong risk factor for chronic pancreatitis. The authors hypothesized that the variant causes impaired trypsin inhibition, which would result in elevated intrapancreatic trypsin activity and pancreatic injury. However, subsequent functional studies demonstrated that the N34S variant had no effect on the trypsin inhibitory activity, expression, or secretion of SPINK1 (5, 6, 7, 8, 9, 10, 11, 12). Despite the accumulating negative evidence, some authors continued to propose that defective trypsin binding must underlie the pathogenic mechanism of the N34S variant and called for more detailed biochemical analysis (11, 13).

The discovery of additional SPINK1 variants associated with chronic pancreatitis confirmed that loss of function is indeed the mechanism responsible for the increased disease risk. Among these, the relatively frequent splice-site variant c.194+2T>C offered the best evidence, as the strong genetic association was complemented with conclusive functional data demonstrating exon skipping and loss of SPINK1 expression (10, 14, 15). Other loss-of-function variants reported include promoter variants, splice-site variants, nucleotide insertion or deletions, loss of the initiator methionine codon, signal peptide variants that reduce secretion, and missense variants that diminish SPINK1 secretion presumably because of misfolding and intracellular retention ([2] and references therein). Remarkably, the large majority of SPINK1 variants seem to affect expression/secretion of the inhibitor rather than its trypsin inhibitory activity. To date, no systematic study analyzed whether any of the reported missense SPINK1 variants affect trypsin binding and inhibition. Human trypsins are post-translationally sulfated on Tyr154, and this modification alters the S2' binding subsite (Schechter–Berger nomenclature) (16, 17, 18, 19). Therefore, to obtain meaningful results, we performed SPINK1 binding studies with sulfated human trypsins. We included both cationic trypsin and anionic trypsin in the analysis, which constitute at least 95% of digestive trypsins. The minor isoform mesotrypsin is resistant to SPINK1 inhibition and was not studied for binding (20, 21). First, we surveyed all reported missense SPINK1 variants and identified those with preserved secretion. Next, we purified these variants and performed quantitative binding studies with human trypsins.

## Results

### Missense SPINK1 variants with preserved secretion

**Figure 1:**
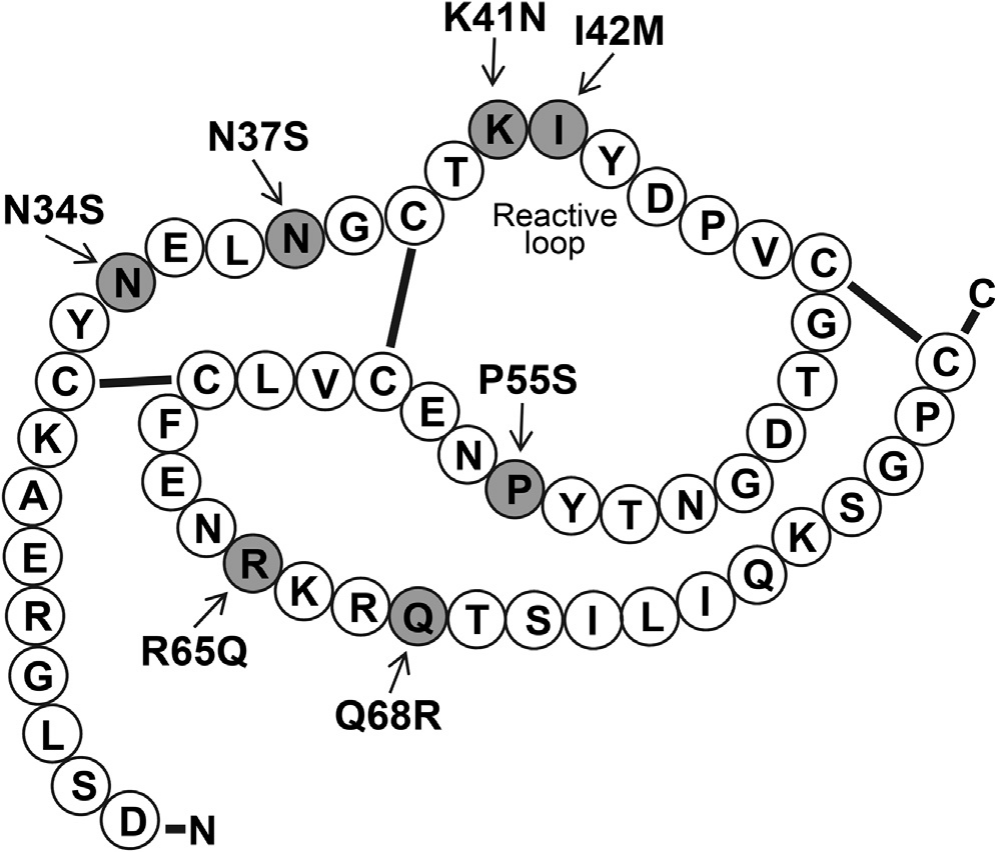
**SPINK1 inhibitor variants with preserved secretion in chronic pancreatitis.** Primary structure of SPINK1. The positions of the investigated mutations are highlighted in *gray*. SPINK1, serine protease inhibitor Kazal type 1.

We found 19 missense variants reported in the literature that introduced single amino acid changes in the mature SPINK1 protein (Table 1). For this study, missense variants that affected the secretory signal peptide were excluded as these cannot alter binding properties of SPINK1 (22). We and others previously assessed the cellular expression and secretion of 14 of the 19 variants in transiently transfected cells (7, 8, 23, 24), and now, we analyzed the remaining five, K41N, I42M, P45S, V46D, and R65W. The results demonstrated that only seven of the 19 SPINK1 variants were secreted to the conditioned medium, including the newly tested variants K41N and I42M. All other variants, including P45S, V46D, and R65W, exhibited defective secretion, in all likelihood because of misfolding and intracellular retention (not shown). Position of the variants with preserved secretion in the SPINK1 primary structure is shown in Figure 1.

**Table 1:** Missense mutations in mature SPINK1 identified in patients with CP (CP carriers) and individuals without CP (non-CP carriers)

Exon	Nucleotide change	Amino acid change	CP carriers reported	Non-CP carriers reported	Carrier frequency in gnomAD
**Exon 3**	**c.101A>G**	**p.N34S**	**1889 (238 hm)**	**402 (9 hm)**	**1.8%**
**Exon 3**	**c.110A>G**	**p.N37S**	**3**		**0.04%**
**Exon 3**	**c.123G>C**	**p.K41N**	**1**	**1**	Not reported
**Exon 3**	**c.126A>G**	**p.I42M**	**1**		**0.006%**
Exon 3	c.133C>T	p.P45S	2		Not reported
Exon 3	c.137T>A	p.V46D	1		Not reported
Exon 3	c.143G>A	p.G48E	1	1	Not reported
Exon 3	c.150T>G	p.D50E	1		0.0004%
Exon 3	c.160T>C	p.Y54H	2		Not reported
**Exon 3**	**c.163C>T**	**p.P55S**	**50 (1 hm)**	**57 (1 hm)**	**0.9%**
Exon 3	c.190A>G	p.N64D	2		Not reported
Exon 3	c.193C>T	p.R65W	1		0.003%
**Exon 3**	**c.194G>A**	**p.R65Q**	**6 (1 hm)**	**2**	**0.1%**
Exon 4	c.198A>C	p.K66N	2		0.023%
Exon 4	c.199C>T	p.R67C	4	2	0.003%
Exon 4	c.200G>A	p.R67H	15	1	0.32%
**Exon 4**	**c.203A>G**	**p.Q68R**	**1**		**0.02%**
Exon 4	c.206C>T	p.T69I	1		0.001%
Exon 4	c.236G>T	p.C79F	1		Not reported

CP, chronic pancreatitis.

Missense variants in exon 1 that affect the secretory signal peptide were excluded. The emboldened variants with preserved secretion were analyzed in this study. The number of homozygous (hm) carriers within the total number is indicated in parenthesis. Data were obtained from the pancreasgenetics.org and gnomad.broadinstitute.org Web sites on July 9, 2020.

**Figure 2:**
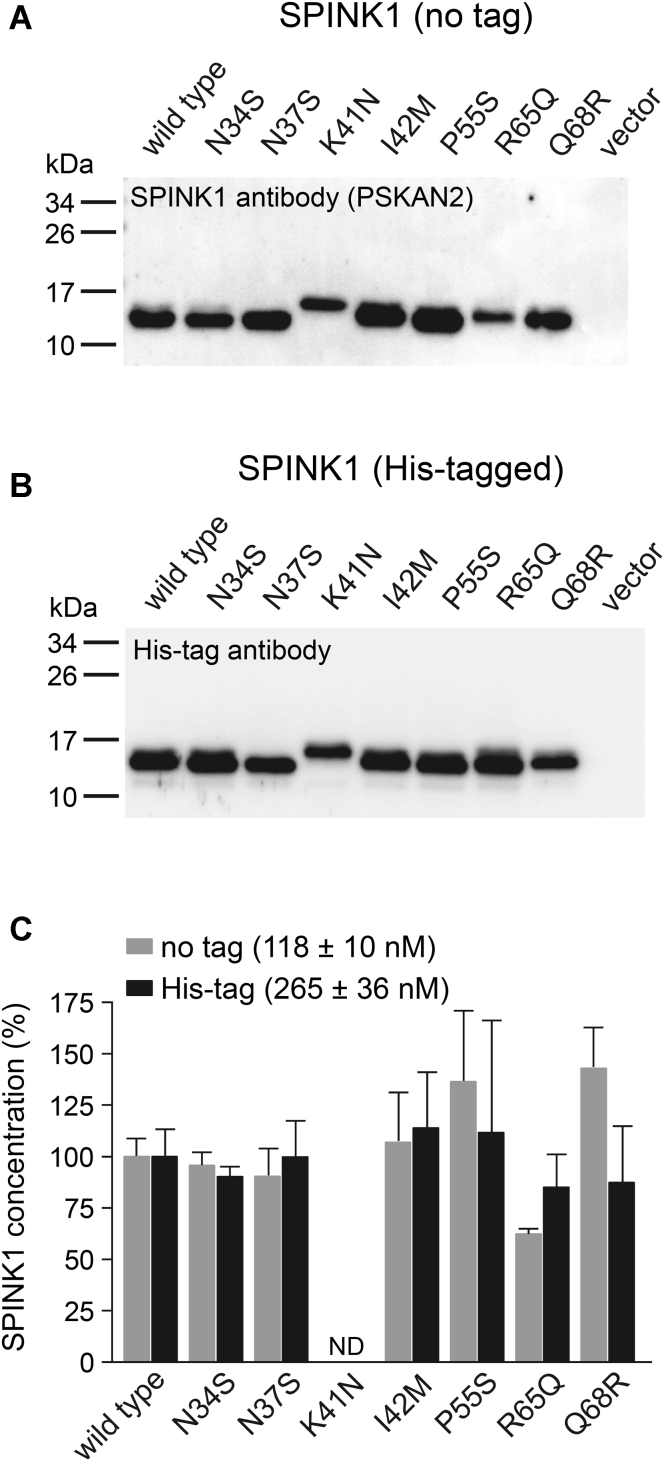
**Secretion of SPINK1 inhibitor variants from transfected human embryonic kidney (HEK) 293T cells.***A*, secretion of untagged wildtype SPINK1 and indicated variants. *B*, secretion of His-tagged wildtype SPINK1 and indicated variants. Conditioned media (10 μl) were analyzed by 15% SDS-PAGE and Western blotting using an anti-SPINK1 antibody (PSKAN2) or an anti–His-tag antibody, as described in Experimental procedures section. *C*, trypsin inhibitory activity of conditioned media from HEK 293T cells transfected with untagged (*gray bars*) or His-tagged (*black bars*) wildtype SPINK1 and the indicated variants. SPINK1 concentrations were determined by titration against human cationic trypsin, as described in Experimental procedures section. Relative SPINK1 levels were expressed as percent of the wildtype protein. The absolute concentrations of the untagged and His-tagged wildtype proteins are also indicated (mean ± SD, n = 4). ND, not determined; levels of variant K41N could not be measured with this assay. Control media from transfections with vector only exhibited low but measurable inhibitory activity (22 ± 2 nM, n = 4), and this background was not subtracted. SPINK1, serine protease inhibitor Kazal type 1.

We characterized secretion of the seven variants from transfected human embryonic kidney 293T (HEK 293T) cells using Western blotting (Fig. 2, *A* and *B*) and by measuring their trypsin inhibitory activity (Fig. 2*C*). For these experiments, we used SPINK1 constructs with and without a C-terminal 10His tag. SPINK1 in the conditioned medium was detected by a monoclonal anti-SPINK1 antibody and by an antibody against the His tag. When untagged SPINK1 constructs were analyzed by Western blotting, secretion of variants K41N and R65Q was somewhat decreased, that of P55S slightly increased, whereas all other variants were secreted at similar levels as the wildtype protein (Fig. 2*A*). Reduced secretion of the R65Q variant was described in prior studies (7, 8, 24); however, the previously reported robust secretion increase of variant Q68R (23) could not be replicated. Western blotting of His-tagged SPINK1 constructs revealed a similar banding pattern, with the exception of variant R65Q, which showed improved secretion (Fig. 2*B*). Finally, we estimated SPINK1 levels by measuring the trypsin inhibitory activity of the conditioned medium (Fig. 2*C*). Relative SPINK1 concentrations in this assay showed good agreement with the Western blot results, with the sole exception of the untagged Q68R variant, which yielded higher than expected concentrations. We also noted that His tagging increased SPINK1 secretion by more than twofold. Taken the results together, we conclude that all seven SPINK1 variants studied are secreted well from transfected cells, and differences in secretion efficiency among the variants were relatively small.

### Modeling the effect of SPINK1 variants on trypsin inhibition

**Figure 3:**
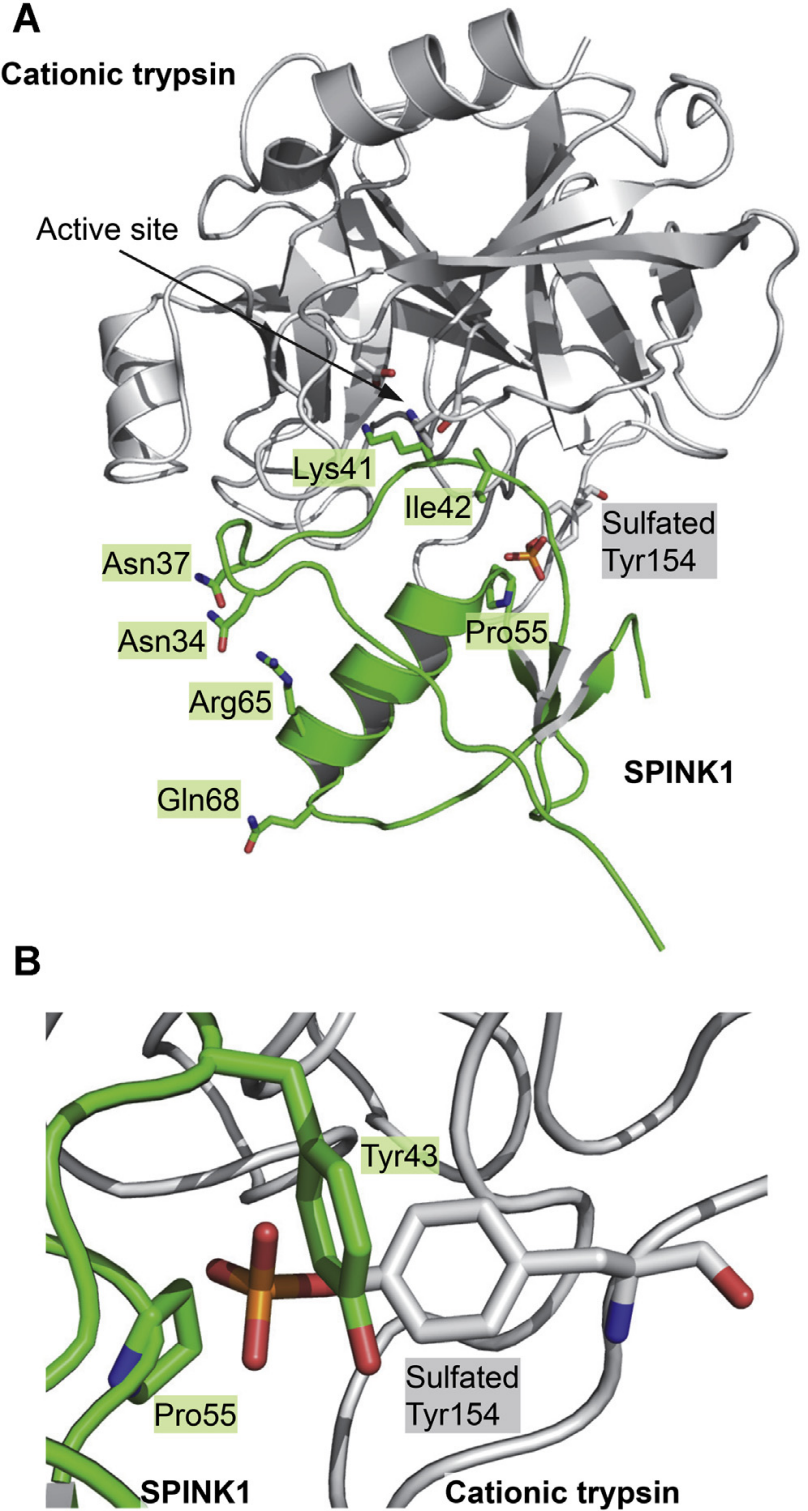
**Interaction of SPINK1 inhibitor with sulfated human cationic trypsin.***A*, ribbon diagram of SPINK1 inhibitor in complex with sulfated cationic trypsin. Positions of the amino acids mutated in the SPINK1 variants are indicated. The model was created by sequence alignment and structural superposition of an engineered SPINK1 variant in complex with chymotrypsinogen A (Protein Data Bank ID: 1CGI) and sulfated cationic trypsin (Protein Data Bank ID: 1TRN) using PyMOL 2.4. The reactive-site peptide bond residues were restored to Lys41 and Ile42. *B*, interactions of SPINK1 with the sulfated Tyr154 residue in cationic trypsin. The sulfate group is in close proximity to SPINK1 residues Tyr43 and Pro55. SPINK1, serine protease inhibitor Kazal type 1.

There are no X-ray structures of human SPINK1 bound to a human trypsin. Therefore, we built a model by superimposing the structure of native sulfated human cationic trypsin onto bovine chymotrypsinogen A complexed with an artificial K41Y,I42E SPINK1 variant, and we restored the reactive-site peptide bond residues to Lys41–Ile42 (25, 26, 27). Examination of the amino acid side chains affected by the seven secreted SPINK1 variants indicated that mutations K41N and I42M alter the Lys41–Ile42 reactive-site peptide bond (positions P1–P1' in the Schechter–Berger numbering of scissile peptide bonds) and likely cause binding impairment (Fig. 3*A*). In contrast, the variants N34S, N37S, R65Q, and Q68R affect side chains that do not directly interact with trypsin and, therefore, are less likely to disturb inhibitor binding. Interestingly, in our putative model, the sulfate group on Tyr154 of trypsin lies in proximity of Pro55, suggesting that variant P55S may influence inhibitor binding to sulfated trypsins (Fig. 3*B*). Furthermore, the Tyr43 side chain of SPINK1, which corresponds to the P2' position in the reactive loop, also appears to sterically clash with the sulfate group, suggesting that SPINK1 might bind weaker to sulfated trypsins than to nonsulfated trypsins. In the following experiments, we tested these predictions.

### Binding of wildtype SPINK1 and the N34S variant to human trypsins

**Figure 4:**
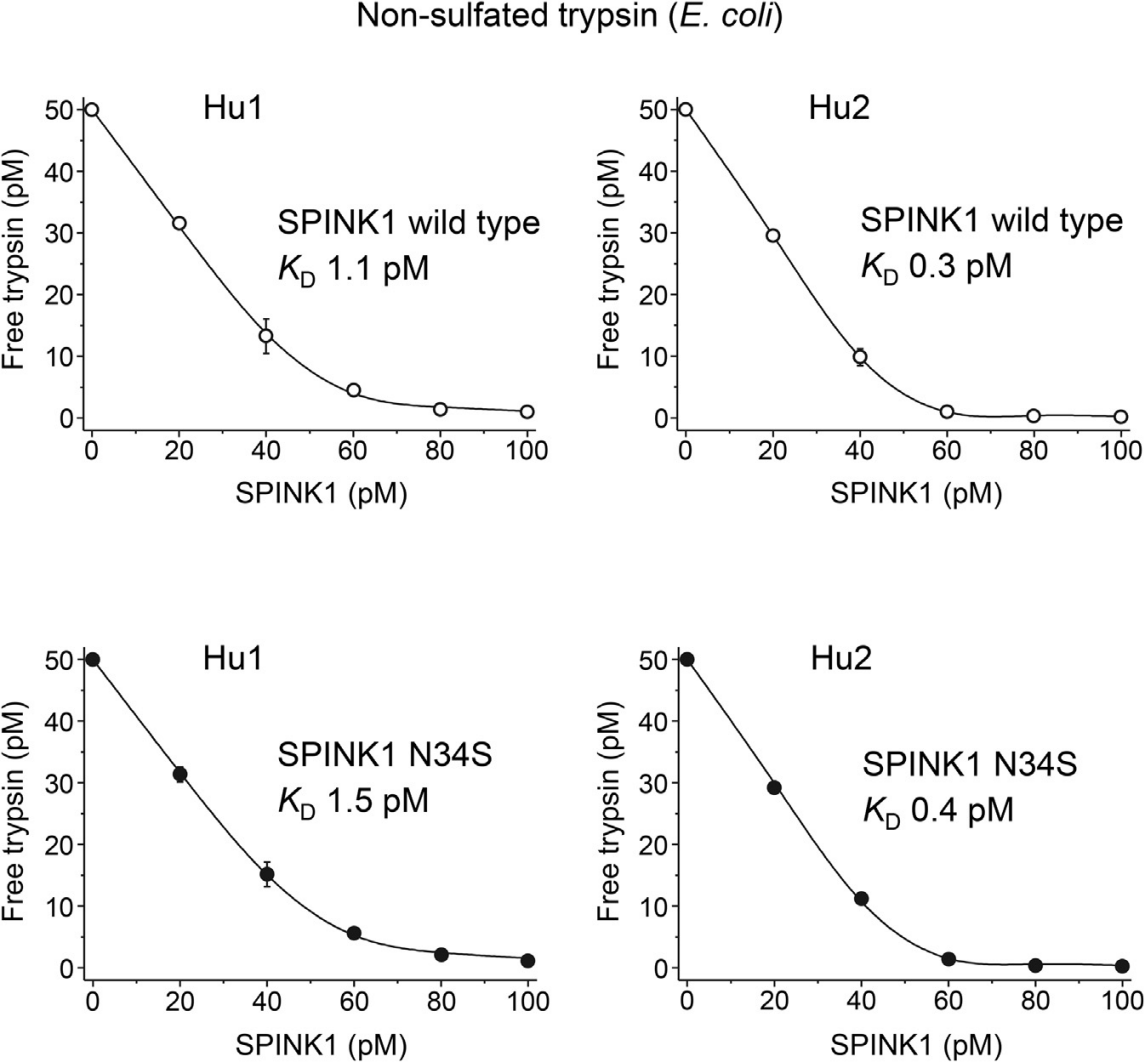
**Inhibition of nonsulfated human trypsins by wildtype SPINK1 and variant N34S.** Binding of SPINK1 to nonsulfated human cationic (Hu1) and anionic (Hu2) trypsin isoforms (purified from *Escherichia coli*) was characterized by determining the dissociation constant (*K*_*D*_) values in equilibrium. Measurements were carried out as described in Experimental procedures section. Data from three experiments were fitted globally. The *K*_*D*_ values are indicated. Data points represent mean ± SD. Symbol size may be larger than the error bars. SPINK1, serine protease inhibitor Kazal type 1.

**Figure 5:**
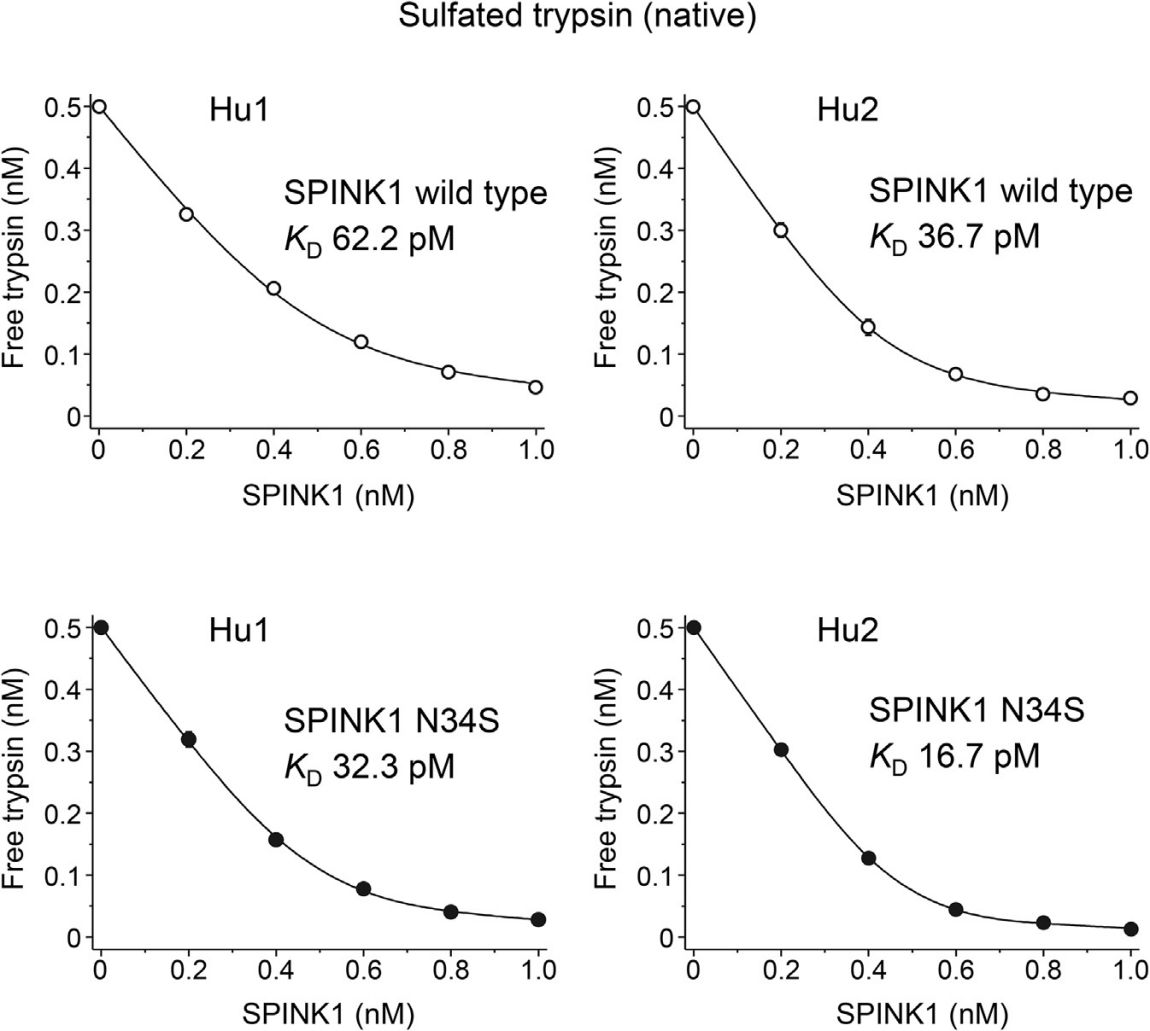
**Inhibition of sulfated human trypsins by wildtype SPINK1 and variant N34S.** Binding of SPINK1 to native sulfated cationic (Hu1) and anionic (Hu2) trypsin isoforms (purified from pancreatic juice) was characterized by determining the dissociation constant (*K*_*D*_) values in equilibrium. Measurements were carried out as described under Experimental procedures section. Data from three experiments were fitted globally. The *K*_*D*_ values are indicated. Data points represent mean ± SD. Symbol size may be larger than the error bars. SPINK1, serine protease inhibitor Kazal type 1.

Since the contentious question remains whether variant N34S alters inhibitor binding, we performed a comprehensive analysis of this variant. For these experiments, we used purified His-tagged SPINK1 proteins, as described in Experimental procedures section. Prior work demonstrated that inhibition of trypsin by wildtype SPINK1 and the N34S variant was comparable (5, 6, 7); however, these studies had technical limitations, as discussed later. First, we measured equilibrium binding to recombinant trypsins produced in *Escherichia coli*, which are not sulfated (Table 2). As shown in Figure 4, strong binding of wildtype SPINK1 to cationic and anionic trypsins was observed with *K*_*D*_ values of 1.1 and 0.3 pM, respectively. Similar *K*_*D*_ values (1.5 and 0.4 pM, respectively) were observed for the N34S variant too (Fig. 5). As predicted by our modeling, when binding was tested on sulfated trypsins that were purified from pancreatic juice, the *K*_*D*_ values were considerably higher (Table 2). Thus, wildtype SPINK1 inhibited native cationic and anionic trypsins with *K*_*D*_ values of 62.2 and 36.7 pM, respectively, whereas the N34S variant exhibited *K*_*D*_ values of 32.3 and 16.7 pM (Figs. 4 and 5). The observations indicate that trypsin sulfation has a highly significant impact on inhibitor binding. Furthermore, the N34S variant inhibited trypsins slightly better, although this difference was within experimental error. Nonetheless, we can conclusively rule out that impaired trypsin inhibition might be caused by the N34S variant.

**Table 2:** Equilibrium dissociation constant (*K*_*D*(eq)_) values and association (*k*_on_) and dissociation (*k*_off_) rate constants determined for wildtype and N34S variant SPINK1 inhibitors against nonsulfated (purified from *Escherichia coli*) and native sulfated (purified from pancreatic juice) human trypsins

SPINK1	Hu1 (*E. coli*)	Hu1-SO_4_ (native)	Hu2 (*E. coli*)	Hu2-SO_4_ (native)
Wildtype				
*k*_on_ (×10^6^ M^−1^s^−1^)	3.0 ± 0.3	3.5 ± 0.2	3.8 ± 0.2	2.4 ± 0.1
*k*_off_ (×10^−6^ s^−1^)	8.2 ± 0.3	54.8 ± 6.2	0.5 ± 0.04	18.1 ± 0.8
*K*_*D*(calc)_ (pM)	2.7	15.8	0.1	7.5
*K*_*D*(eq)_ (pM)	1.1 ± 0.2	62.2 ± 10.3	0.3 ± 0.02	36.7 ± 2.3
N34S				
*k*_on_ (×10^6^ M^−1^s^−1^)	5.3 ± 0.2	2.7 ± 0.2	5.1 ± 0.2	3.1 ± 0.3
*k*_off_ (×10^-6^ s^−1^)	7.0 ± 0.6	45.0 ± 5.1	0.4 ± 0.01	13.6 ± 0.5
*K*_*D*(calc)_ (pM)	1.3	16.4	0.1	4.4
*K*_*D*(eq)_ (pM)	1.5 ± 0.2	32.3 ± 2.9	0.4 ± 0.1	16.7 ± 0.8

Hu1, nonsulfated human cationic trypsin; Hu2, nonsulfated human anionic trypsin; Hu1-SO_4_, native sulfated human cationic trypsin; Hu2-SO_4_, native sulfated human anionic trypsin; *K*_*D*(calc)_, equilibrium dissociation constant calculated from the rate constants.

Measurements were carried out as described under Experimental procedures section. Data from at least three experiments were fitted globally. The error of the fit is indicated.

In biological systems, rate of association or dissociation of the inhibitor may be more relevant functionally than the equilibrium binding strength. Therefore, we measured the *k*_on_ and *k*_off_ rate constants for wildtype and N34S SPINK1 against nonsulfated and sulfated human trypsins, as detailed in Experimental procedures section and illustrated in Figures S1 and S2. As shown in Table 2, similar values were obtained for the two inhibitors. When nonsulfated and sulfated trypsins were compared, SPINK1 associated slightly faster with the nonsulfated forms, whereas dissociation was significantly more rapid from the sulfated forms. Overall, the increased dissociation explains the higher *K*_*D*_ values observed in equilibrium binding experiments. We calculated *K*_*D*_ values from the rate constants and compared them with those obtained in the equilibrium binding assays. There was good agreement on nonsulfated trypsins where all constants were picomolar or lower. We note, however, that the experimental system used is not reliable in detecting differences in the subpicomolar *K*_*D*_ range. With respect to sulfated and native trypsins, the calculated *K*_*D*_ values were about two- to fivefold lower than those measured directly. This variation is within acceptable limits, given that two experimentally different methods were used to determine the *K*_*D*_ values.

### Temporary inhibition by wildtype SPINK1 and the N34S mutant

**Figure 6:**
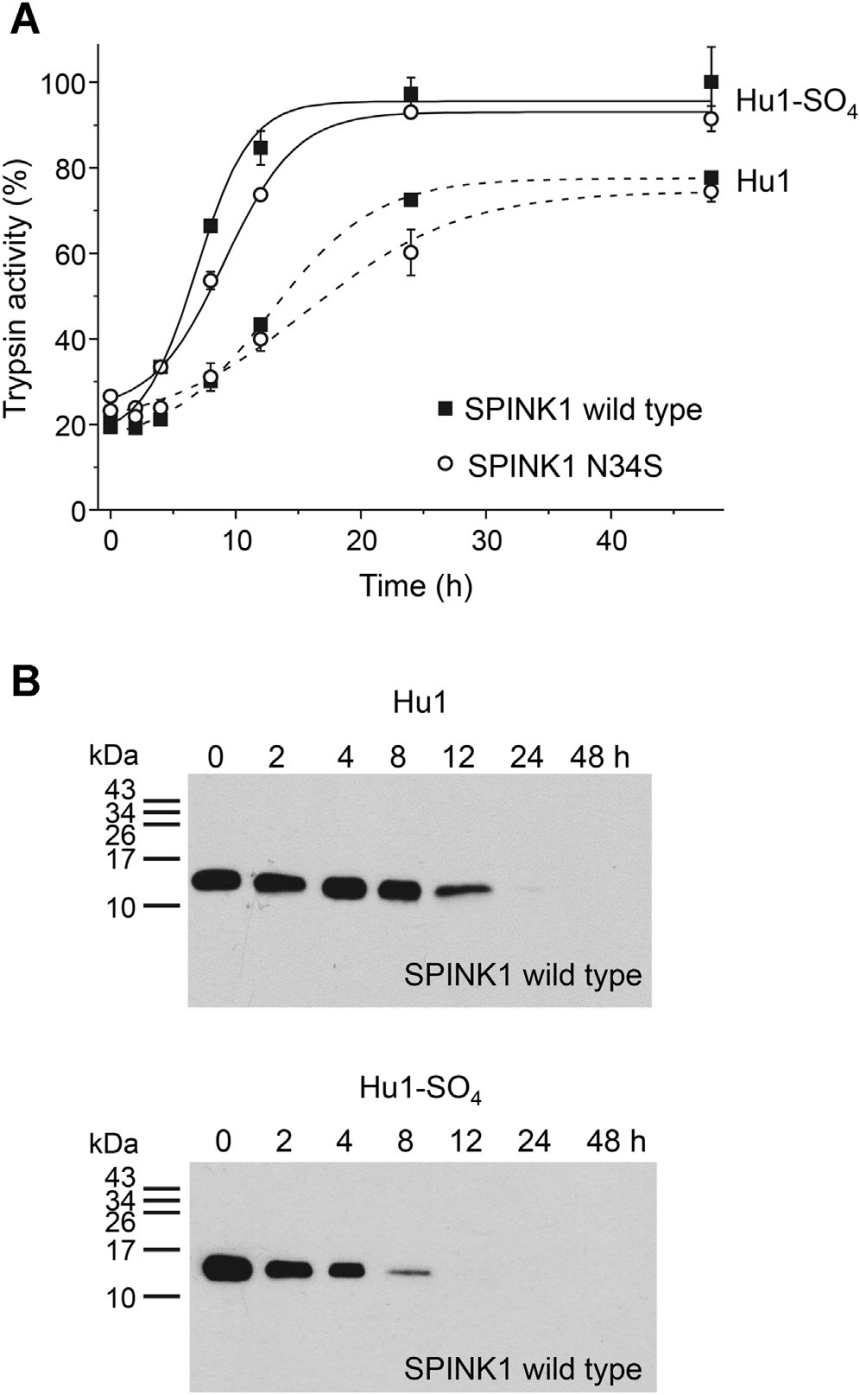
**Temporary inhibition of human cationic trypsin by wildtype SPINK1 and variant N34S.** SPINK1 (25 nM) was incubated with nonsulfated (Hu1) or sulfated (Hu1-SO_4_) cationic trypsin (30 nM) for 30 min, in 0.1 M Tris–HCl (pH 8.0) buffer supplemented with 1 mM CaCl_2_ and 0.05% Tween 20 at 23 °C to achieve 80% decrease of trypsin activity. *A*, at the indicated times, 100 μl aliquots were withdrawn, and trypsin activity was measured by the addition of 2.5 μl 6 mM Suc-Ala-Ala-Pro-Lys-*p*-nitroanilide substrate. Trypsin activity is expressed as a percentage of the maximal activity measured in the absence of SPINK1. Data points represent mean ± SD. Symbol size may be larger than the error bars. *B*, at the indicated times, 200 μl aliquots were precipitated with 10% trichloroacetic acid and analyzed by 18% SDS-PAGE and Western blotting using an anti-His tag antibody, as described in Experimental procedures section. Hu1, nonsulfated cationic trypsin expressed in *Escherichia coli*; Hu1-SO_4_, sulfated cationic trypsin purified from pancreatic juice; SPINK1, serine protease inhibitor Kazal type 1.

SPINK1 is a so-called temporary inhibitor as it becomes inactivated over time and releases the active protease (28). This process starts with the cleavage of the SPINK1 reactive-site peptide bond by the bound trypsin followed by inactivating cleavages at Arg67–Gln68 and elsewhere by excess trypsin (20, 29, 30, 31). We compared the release of nonsulfated and sulfated human cationic trypsin from SPINK1 complexes and found that sulfated trypsin was released at a significantly higher rate than nonsulfated trypsin (Fig. 6*A*). This can be best explained by the more rapid dissociation of SPINK1 from sulfated trypsin (Table 2), which also underlies the elevated *K*_*D*_ value of the interaction. Western blotting confirmed time-dependent SPINK1 degradation that paralleled the liberation of trypsin activity (Fig. 6*B*). As expected, the kinetics of temporary inhibition were similar for wildtype and N34S SPINK1, although we noted a slightly slower release for the N34S variant.

### Trypsin sulfation weakens SPINK1 binding

An original observation of the present study was that sulfation of human trypsins on Tyr154 weakens binding of SPINK1 and facilitates its release from the complex. Since our nonsulfated and sulfated trypsin preparations were derived from different sources (*E. coli versus* pancreatic juice), we wanted to rule out any confounding effects this may have caused. Therefore, we expressed human cationic and anionic trypsins in HEK 293T cells in nonsulfated and sulfated forms, as described in Experimental procedures section. We purified the nonsulfated and sulfated trypsins from the conditioned medium and performed equilibrium binding assays with wildtype and N34S SPINK1. As shown in Table 3, the *K*_*D*_ values were consistently higher against sulfated trypsins *versus* nonsulfated trypsins, confirming the weakening effect of trypsin sulfation on SPINK1 binding. Interestingly, when compared with values obtained on trypsin preparations from *E. coli* and pancreatic juice (Table 2), the *K*_*D*_ values of nonsulfated trypsins from HEK 293T cells were somewhat higher, whereas those of sulfated trypsins from HEK 293T cells were mostly lower. As a result, the negative effect of trypsin sulfation on SPINK1 binding appeared smaller in these experiments. While the reason for this variability is not readily apparent, it does not change the conclusion that trypsin sulfation weakens SPINK1 binding.

**Table 3:** Equilibrium dissociation constant (*K*_*D*_) values determined for wildtype and N34S variant SPINK1 inhibitors against nonsulfated and sulfated human trypsins purified from the conditioned medium of transfected HEK 293T cells

*K*_*D*_ (pM)	Hu1 (HEK 293T)	Hu1-SO_4_ (HEK 293T)	Hu2 (HEK 293T)	Hu2-SO_4_ (HEK 293T)
SPINK1 wildtype	4.4 ± 0.4	40.2 ± 7.8	0.5 ± 0.2	9.0 ± 1.1
SPINK1 N34S	4.7 ± 0.3	37.5 ± 4.3	0.8 ± 0.2	6.3 ± 0.7

Hu1, nonsulfated human cationic trypsin; Hu2, nonsulfated human anionic trypsin; Hu1-SO_4_, sulfated human cationic trypsin; Hu2-SO_4_, sulfated human anionic trypsin.

Measurements were carried out as described under Experimental procedures section. Data from three experiments were fitted globally. The error of the fit is indicated.

### Role of Tyr43 in SPINK1 binding to human trypsins

Modeling suggested that steric clash of Tyr43 with the sulfate group might be responsible for the impaired SPINK1 binding to sulfated trypsins (Fig. 3). To test this notion, we mutated Tyr43 in SPINK1 to Ala and Arg and tested equilibrium binding to nonsulfated and sulfated cationic trypsins (Table 4). Compared with wildtype SPINK1, mutants Y43A and Y43R showed almost 20-fold and 100-fold reduced binding to nonsulfated cationic trypsin, respectively. In contrast, binding to sulfated cationic trypsin was comparable for wildtype and Y43A inhibitors, whereas mutant Y43R showed threefold improved binding. The decreased binding of the Y43A mutant to nonsulfated trypsin argues that Tyr43 participates in an important stabilizing interaction, possibly stacking against Tyr154 on trypsin. Since the same interaction is absent when bound to sulfated trypsin, *K*_*D*_ values for wildtype and Y43A mutant SPINK1 are similar. Mutation Y43R results in unfavorable interactions with nonsulfated trypsin, whereas it improves binding to sulfated trypsin, likely through an electrostatic interaction between the guanidinium and sulfate groups. Taken together, the discrepant effects of the Tyr43 mutations are consistent with the notion that this side chain is a key determinant of the weaker SPINK1 binding to sulfated trypsins.

**Table 4:** Equilibrium dissociation constant (*K*_*D*_) values determined for wildtype and Y43A and Y43R mutant SPINK1 inhibitors against nonsulfated and sulfated human cationic trypsins

*K*_*D*_ (pM)	Hu1 (*Escherichia coli*)	Hu1-SO_4_ (native)
SPINK1 wildtype	1.1 ± 0.4	67.4 ± 7.7
SPINK1 Y43A	20.3 ± 2.5	62.2 ± 5.6
SPINK1 Y43R	103.8 ± 13.9	27.1 ± 2.3

Hu1, nonsulfated human cationic trypsin purified from *E. coli*; Hu1-SO_4_, native sulfated human cationic trypsin purified from pancreatic juice.

Measurements were carried out as described under Experimental procedures section. Data from three experiments were fitted globally. The error of the fit is indicated.

### Binding of missense SPINK1 variants to native human trypsins

**Figure 7:**
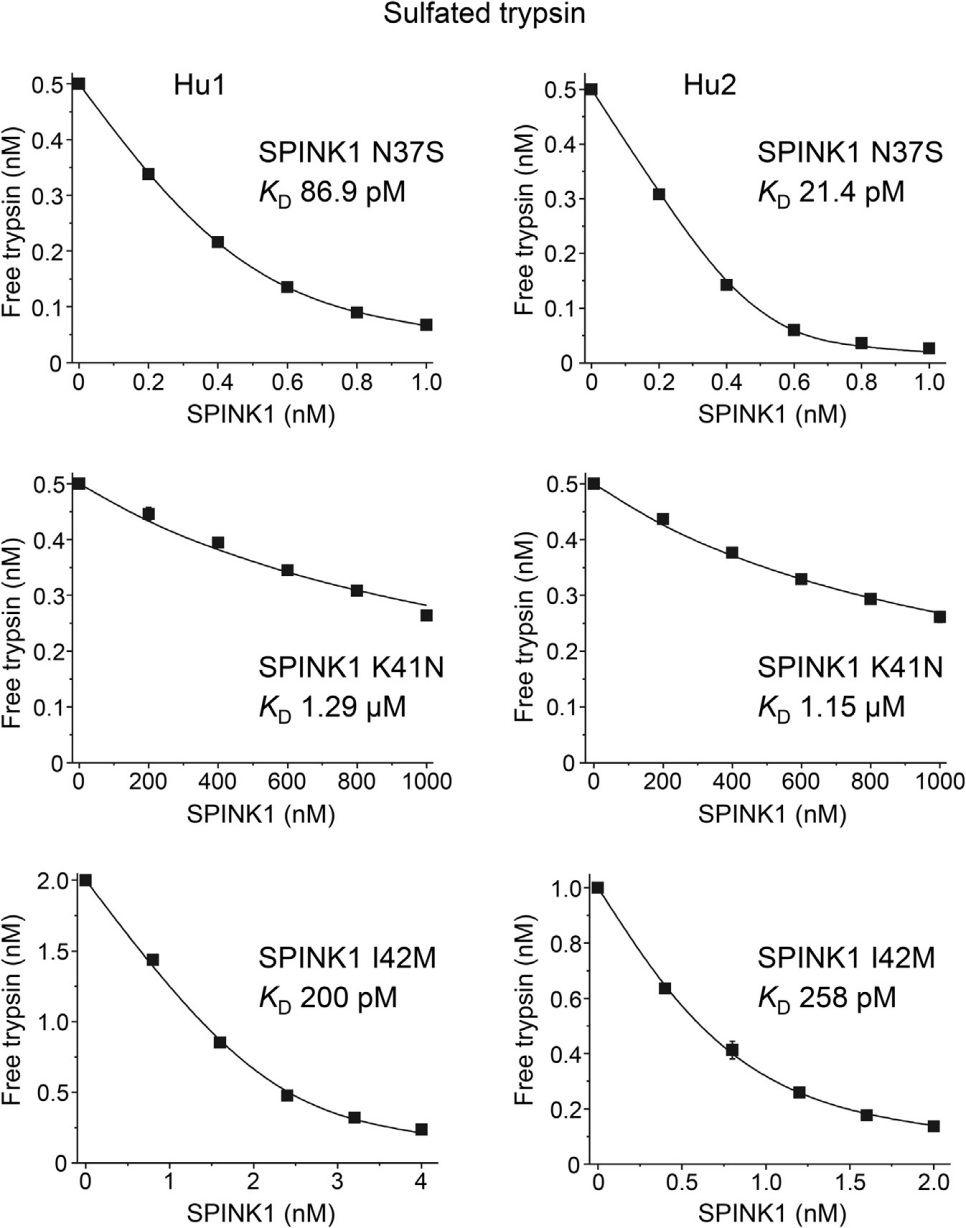
**Inhibition of human trypsins by SPINK1 variants N37S, K41N, and I42M.** Binding of SPINK1 to sulfated cationic (Hu1) and anionic (Hu2) trypsins purified from pancreatic juice was characterized by determining the dissociation constant (*K*_*D*_) values in equilibrium. Measurements were carried out as described under Experimental procedures section. Data from three experiments were fitted globally. The *K*_*D*_ values are indicated. Data points represent mean ± SD. Symbol size may be larger than the error bars. SPINK1, serine protease inhibitor Kazal type 1.

**Figure 8:**
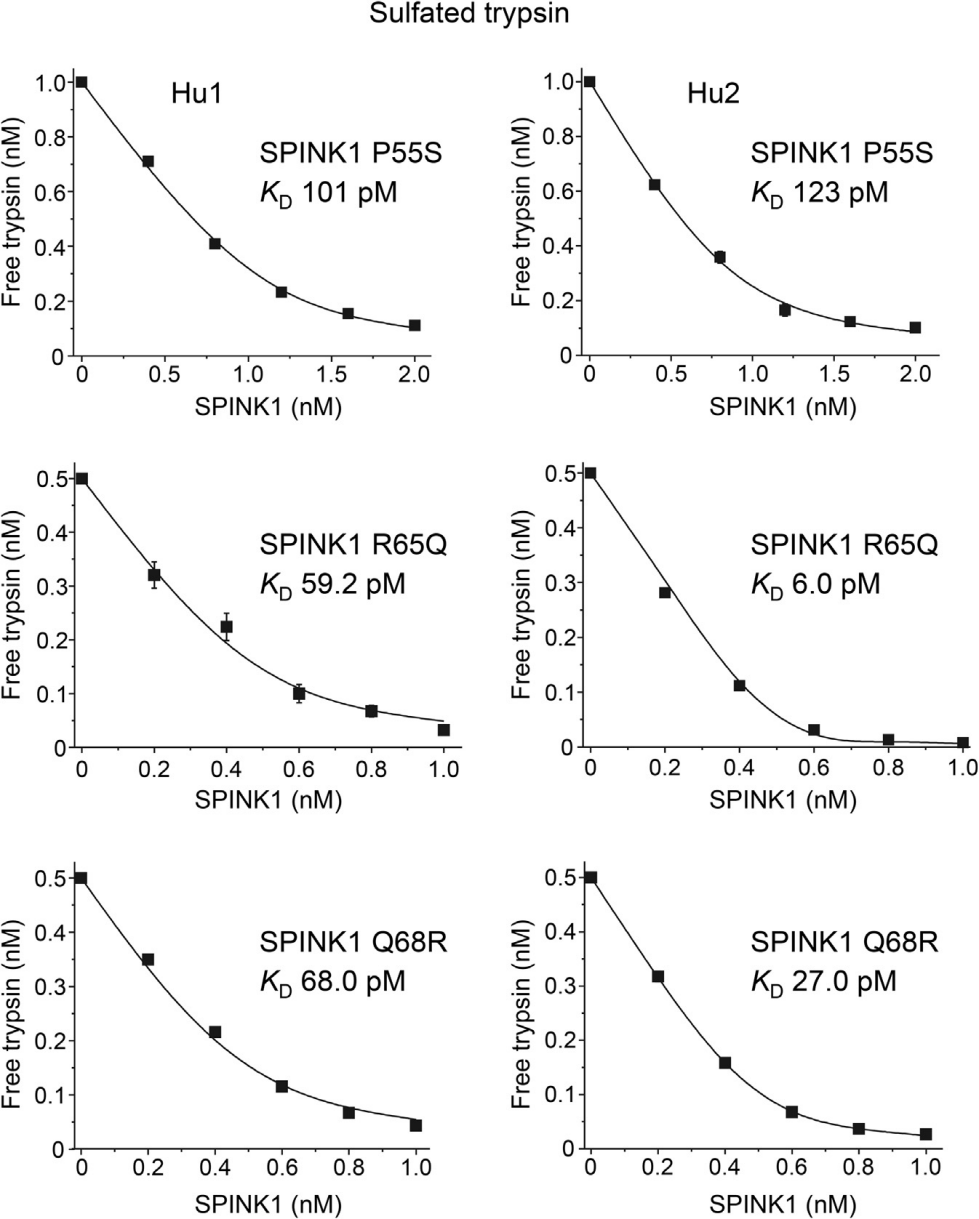
**Inhibition of human trypsins by SPINK1 variants P55S, R65Q, and Q68R.** Binding of SPINK1 to sulfated cationic (Hu1) and anionic (Hu2) trypsins purified from pancreatic juice was characterized by determining the dissociation constant (*K*_*D*_) values in equilibrium. Measurements were carried out as described under Experimental procedures section. *K*_*D*_ values are indicated. Data from three experiments were fitted globally. The *K*_*D*_ values are indicated. Data points represent mean ± SD. Symbol size may be larger than the error bars. SPINK1, serine protease inhibitor Kazal type 1.

**Figure 9:**
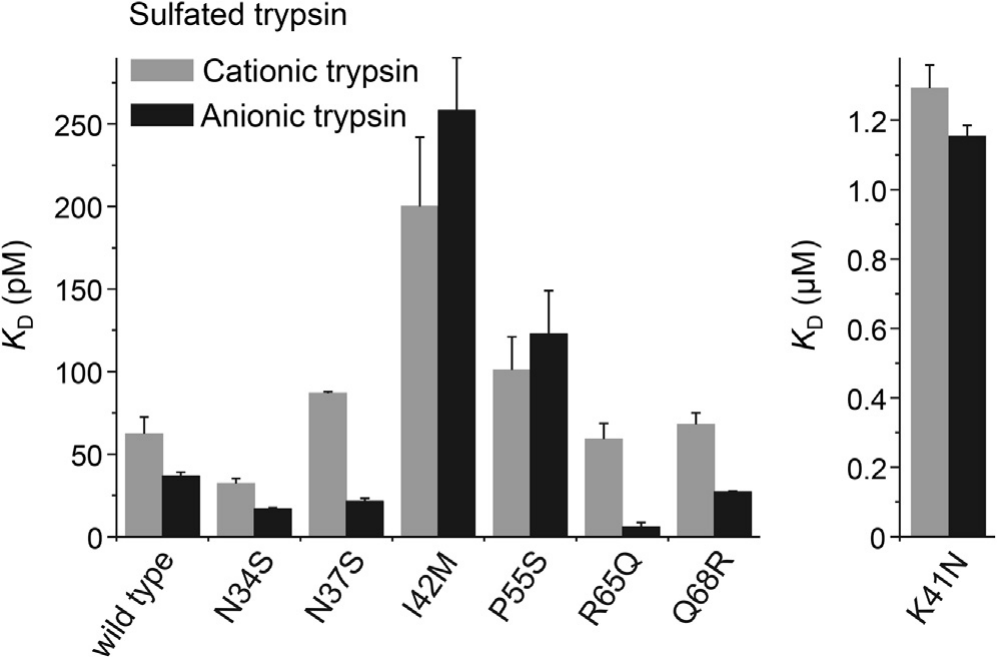
**Equilibrium dissociation constants of wildtype SPINK1 and seven variants with sulfated human trypsins.** Dissociation constant values (*K*_*D*_) were determined in equilibrium, as described in Experimental procedures section. Sulfated trypsins were purified from pancreatic juice. Data from three experiments were fitted globally. Error bars indicate the errors of the fits. See Table 5 for exact values. SPINK1, serine protease inhibitor Kazal type 1.

In addition to N34S, we purified six other His-tagged SPINK1 variants that showed preserved secretion (Fig. 2). To characterize their inhibitory activity, we measured equilibrium binding to sulfated human cationic and anionic trypsins purified from pancreatic juice. From a disease perspective, these are the pathologically relevant interactions that need to be characterized. The binding experiments are shown in Figures 7 and 8, and the *K*_*D*_ values are summarized in Table 5 and Figure 9. As expected, mutant K41N bound poorly to the two trypsins, with micromolar *K*_*D*_ values, that corresponded to a 20,000- to 30,000-fold reduction in affinity. The reactive-site mutant I42M reduced binding by three- to sevenfold. Mutation P55S caused a slight decrease in binding, by 1.6- to 3.4-fold, which was almost within experimental error. Finally, SPINK1 variants N34S, N37S, R65Q, and Q68R exhibited *K*_*D*_ values that were similar to or even smaller than those of wildtype SPINK1. Taken together, only rare SPINK1 variants K41N and I42M that directly affect the reactive-site peptide bond compromised inhibitor binding to a significant extent.

**Table 5:** Equilibrium dissociation constant (*K*_*D*_) values determined for wildtype SPINK1 and seven variants on sulfated cationic and anionic trypsin isoforms

*K*_*D*_ (pM)	Hu1-SO_4_	Hu2-SO_4_
SPINK1 wildtype	62.2 ± 10.3	36.7 ± 2.3
SPINK1 N34S	32.3 ± 2.9	16.7 ± 0.8
SPINK1 N37S	86.9 ± 1.0	21.4 ± 1.9
SPINK1 I42M	200 ± 42	258 ± 32
SPINK1 P55S	101 ± 20	123 ± 26
SPINK1 R65Q	59.2 ± 9.4	6.0 ± 2.5
SPINK1 Q68R	68.0 ± 7.1	27.0 ± 0.6
***K***_***D***_**(μM)**		
SPINK1 K41N	1.29 ± 0.07	1.15 ± 0.03

Hu1-SO_4_, sulfated human cationic trypsin purified from pancreatic juice; Hu2-SO_4_, sulfated human anionic trypsin purified from pancreatic juice.

Measurements were carried out as described under Experimental procedures section. Data from three experiments were fitted globally. The error of the fit is indicated.

## Discussion

In the present study, we performed a comprehensive and quantitative functional analysis of all secreted SPINK1 variants with respect to their trypsin inhibitory activity. The issue whether some of these variants exert a pathogenic effect in chronic pancreatitis because of impaired binding to trypsin has been debated as convincing evidence has been lacking. Previously, we and others reported preliminary binding studies for variants N34S, P55S, and R65Q; however, the methodology used was semiquantitative at best (5, 6, 7). Furthermore, these prior studies used either nonsulfated recombinant human cationic trypsin, bovine trypsin, or a commercial human trypsin of unspecified nature. Unlike bovine or most other mammalian trypsins, human trypsins are sulfated on Tyr154, which can affect substrate and inhibitor binding (16, 17, 18, 19). Here, we measured binding of SPINK1 variants to native human cationic and anionic trypsins, which constitute more than 95% of total pancreatic trypsins. The minor human isoform mesotrypsin is poorly inhibited by SPINK1 and was not studied for binding (20, 21). To establish the significance of sulfation, wildtype SPINK1 and the N34S variant were also assayed with nonsulfated recombinant trypsins from *E. coli* and nonsulfated and sulfated recombinant trypsin preparations from HEK 293T cells.

We found that sulfation increased the *K*_*D*_ of SPINK1 binding to cationic trypsin by more than 50-fold and to anionic trypsin by 120-fold, when nonsulfated trypsin from *E. coli* and sulfated trypsin from pancreatic juice were compared. A smaller but still significant ninefold to 18-fold difference was seen when nonsulfated and sulfated trypsins were obtained from transfected HEK 293T cells. The weaker inhibitor binding to sulfated trypsins was mainly because of the more rapid dissociation of the SPINK1–trypsin complex. Modeling suggested that sulfation changes the interaction between trypsin and Tyr43 of the SPINK1 reactive loop. Mutagenesis of Tyr43 supported this assumption, as the effects of Ala and Arg replacements were dependent on the sulfation status of trypsin. The observations underscore the need to use sulfated trypsin preparations when studying binding of SPINK1 variants.

The binding experiments provided conclusive evidence that the N34S variant has no impact on trypsin inhibition whatsoever. Furthermore, during extended incubations with trypsin, wildtype SPINK1 and the N34S variant were degraded and released at a similar rate (temporary inhibition). Human mesotrypsin was previously shown to degrade SPINK1 (20), and this observation raises the possibility that variant N34S might be degraded differently. However, this is not the case either (Fig. S3). Considering other mechanisms that might explain how the N34S variant increases pancreatitis risk, it is noteworthy that the variant is part of an extended haplotype that includes four intronic variants, which are relatively well characterized by genetic and functional studies. In minigene and full-gene splicing assays in transfected cells, none of the intronic variants had an appreciable effect on SPINK1 expression (10, 11). More recently, pancreatic cancer cell lines heterozygous for the N34S variants were shown to express diminished mRNA levels of the variant allele, and a new upstream variant was identified as part of the haplotype (32). Boulling *et al*. (33) demonstrated that this variant affects an enhancer element and proposed that it might cause diminished SPINK1 mRNA expression. Although not conclusive, emerging evidence seems to suggest that the N34S-associated haplotype reduces protective SPINK1 levels, and the pathogenic culprit may be located in the 5’ upstream region.

We documented binding defects of varying extent for variants K41N, I42M, and P55S. Variants K41N and I42M alter the Lys41–Ile42 reactive-site peptide bond of the inhibitor and were predicted to affect inhibitor binding. Both variants are rare, reported only once in the literature, in pediatric cases of recurrent acute pancreatitis (34, 35). Lys41 is the main specificity residue of SPINK1, which is inserted into the S1 binding pocket of trypsin, where it interacts electrostatically with an Asp residue. In the K41N variant, replacement of Lys41 with the uncharged Asn side chain caused a marked 20,000-fold to 30,000-fold decrease in trypsin binding, which should translate to a significant loss of protective SPINK1 function. Variant I42M is a relatively conservative replacement of the Ile42 side chain, which causes a small but still significant reduction in binding affinity, about threefold against cationic trypsin and sevenfold against anionic trypsin. The index patient also carried other risk variants in the chymotrypsin C (*CTRC*) and cystic fibrosis transmembrane conductance regulator (*CFTR*) genes, which is consistent with the notion that the I42M SPINK1 variant has a lesser effect on disease risk than the K41N variant, and additional risk variants are required for penetrance (35). Finally, variant P55S showed a modest binding defect of 1.6-fold against cationic trypsin and 3.4-fold against anionic trypsin. The proximity of Pro55 to the sulfate group on trypsin supports the notion that this is a true effect. The P55S variant is found with circa 0.9% frequency in the general population, and no significant enrichment was reported in patients with pancreatitis (Table 1). In some studies, however, the variant was found in *trans* with other pathogenic SPINK1 variants (4, 36, 37, 38, 39). Taken together with the binding studies, this raises the possibility that P55S increases pancreatitis risk slightly, in a clinically insignificant manner.

Similarly to N34S, variants N37S, R65Q, and Q68R showed normal binding to human trypsins. Previous studies indicated that variant R65Q was secreted at moderately reduced levels from transfected cells, and in the present study, we confirmed this notion (7, 8, 24). Earlier experiments using complementary DNA and minigene constructs suggested that mRNA stability or splicing may be affected by the R65Q variant, but a more recent study using full-gene expression constructs demonstrated no such defects (7, 8, 12, 24, 40). Thus, taken the available evidence together, the reduced secretion of variant R65Q is more likely related to altered folding. This interpretation is supported by the observation that a C-terminal His tag increased secretion of the variant. Finally, variant Q68R was reported to exhibit markedly increased secretion from transfected cells (23). We could not replicate this finding in the present study.

Taken together, our observations indicate that impaired SPINK1 binding to trypsin is uncommon in chronic pancreatitis, typically associated with rare variants that directly affect the reactive site of the inhibitor. The main pathogenic mechanism of SPINK1 variants appears to be loss of trypsin inhibitory function because of reduced expression and/or secretion. Consistent with this notion, mice and humans with homozygous deletion of the *SPINK1* gene develop severe and early onset pancreatitis that results in trypsin-dependent destruction of the pancreas (41, 42, 43, 44).

## Experimental procedures

### Nomenclature

Nucleotide numbering of SPINK1 starts at the first nucleotide of the ATG translation initiation codon. Amino acid sequence numbering starts with the translation initiator methionine of the primary translation product.

### Expression plasmids

For expression studies, the coding DNA of wildtype SPINK1 and indicated variants were synthesized with or without a C-terminal 10His tag (GenScript) and cloned into the pcDNA3.1(−) plasmid between the XhoI and BamHI restriction sites. For purification experiments, wildtype SPINK1 and indicated variants were cloned into the previously described SPINK1-minigene-1 construct, which contained intron 1 appropriately placed within the SPINK1 coding DNA sequence and a newly added C-terminal 10His tag (10). The construction of expression plasmids for human cationic trypsinogen (pTrapT7-Hu1, pcDNA3.1(-)-Hu1), human anionic trypsinogen (pTrapT7-Hu2, pcDNA3.1(-)-Hu2), and tyrosylprotein sulfotransferase 2 (pcDNA3.1(-)-TPST2) were reported previously (18, 45, 46, 47, 48).

### Cell culture and transfection

HEK 293T cells were cultured in six-well tissue culture plates in 2 ml Dulbecco's modified Eagle medium, 10% fetal bovine serum, 4 mM glutamine, and 1× penicillin/streptomycin in a 5% CO_2_ incubator. At 70 to 90% confluence, the cells were transiently transfected with plasmid DNA using Lipofectamin 2000 (Life Technologies). To prepare the transfection mix, the pcDNA3.1(-) plasmid (4 μg) in 0.25 ml Opti-MEM I reduced serum medium was mixed with 10 μl Lipofectamin 2000 in 0.25 ml Opti-MEM and incubated at room temperature for 20 min. To initiate the transfection, 0.5 ml medium was removed from each well and replaced with the transfection mixture. After 15 h of incubation in a CO_2_ incubator, the medium was removed from the wells, the cells were rinsed with 1.0 ml PBS (pH 7.4), and 1.5 ml fresh Opti-MEM was added. Conditioned medium containing the secreted SPINK1 was harvested after 48 h.

### Western blot analysis of conditioned medium

Aliquots of the medium (10 μl) were supplemented with 8 μl 2× Laemmli sample buffer and 2 μl 1 M dithiothreitol. The samples were then heat denatured at 95 ^o^C for 15 min, electrophoresed on 15% minigels, and the proteins were transferred to a polyvinylidene difluoride membrane. The membrane was blocked with the manufacturer's blocking reagent (0.5% in 1× blocking buffer with 0.1% Tween 20), and an anti-His tag antibody conjugated to horseradish peroxidase (HRP) (penta-His HRP conjugate kit; catalog number 34460; Qiagen) was added at a dilution of 1:2000 for 1 h. HRP activity was detected with the SuperSignal West Pico PLUS chemiluminescent substrate (Thermo Scientific). Alternatively, the polyvinylidene difluoride membrane was blocked with 10% solubilized milk powder for 1 h and incubated with a mouse monoclonal anti-SPINK1 antibody (MoBiTec PSKAN2 antibody; purchased from Boca Scientific) at a dilution of 1:500 overnight. HRP-conjugated antimouse IgG (catalog number HAF007; R&D Systems) was used as secondary antibody at 1:2000 dilution for 1 h. HRP activity was detected by SuperSignal West Femto Maximum Sensitivity chemiluminescent substrate (Thermo Scientific).

### Measuring trypsin inhibitory activity of conditioned medium

The concentration of SPINK1 in the conditioned media from HEK 293T cells was determined by titration against human cationic trypsin. A twofold serial dilution of the medium (50 μl) was prepared with assay buffer (0.1 M Tris–HCl [pH 8.0], 1 mM CaCl_2_, and 0.05% Tween 20), and 50 μl trypsin solution (80 nM in assay buffer) was added to each dilution (100 μl final volume and 40 nM final trypsin concentration). After incubation for 30 min at 23 °C, residual trypsin activity was measured by adding 100 μl of 200 μM Z-Gly-Pro-Arg-*p*-nitroanilide substrate, dissolved in assay buffer. Trypsin activity was plotted as a function of the medium volume in the reaction, and SPINK1 concentrations were calculated from the extrapolated × intercepts of the linear portion of the inhibition curves.

### Expression and purification of SPINK1

HEK 293T cells were grown in a T75 tissue culture flask to 70 to 90% confluence and were transfected with the appropriate expression plasmid DNA and branched polyethyleneimine, as described recently (49). In some experiments, transfections were carried out with Lipofectamin 2000, as described (50). After 15 h incubation in a CO_2_ incubator, cells were rinsed with Opti-MEM, and 20 ml fresh Opti-MEM was added to the flasks. After 48 h of incubation, the conditioned medium was harvested, and 20 ml fresh Opti-MEM was added and collected again after 48 h of incubation. SPINK1 carrying a C-terminal 10His tag was purified with a nickel–nitrilotriacetic acid Superflow Cartridge attached to an FPLC system. The cartridge was equilibrated with NPI-20 (50 mM NaH_2_PO_4_, 300 mM NaCl, 20 mM imidazole, pH 8.0) buffer, and approximately 200 ml of conditioned media were loaded at a flow rate of 2 ml/min. Protein contaminants were removed by washing with 60 ml NPI-20, and SPINK1 was eluted with NPI-250 (50 mM NaH_2_PO_4_, 300 mM NaCl, 250 mM imidazole, pH 8.0) buffer. Fractions containing SPINK1 were pooled and dialyzed against 20 mM Tris–HCl (pH 8.0) or desalted on two serially connected 5 ml HiTrap desalting columns on an FPLC system using 20 mM Tris–HCl (pH 8.0) as equilibration and wash buffer.

### Expression and purification of trypsinogen

Nonsulfated cationic trypsinogen (Hu1) and anionic trypsinogen (Hu2) were expressed in *E. coli* BL21(DE3), refolded *in vitro*, and purified by ecotin affinity chromatography, as described before (51, 52). Sulfated trypsinogens were purified from human pancreatic juice on a MonoQ anion exchanger column followed by ecotin affinity chromatography, as described previously (17, 48). Nonsulfated and sulfated trypsinogens were also produced in HEK 293T cells grown in T75 flasks. To produce nonsulfated trypsinogens, cells were transfected with 10 μg pcDNA3.1(-)-Hu1 or pcDNA3.1(-)-Hu2 plasmid and grown in the presence of 50 mM sodium chlorate that inhibits endogenous tyrosylprotein sulfotransferases. To generate completely sulfated trypsinogens, cells were cotransfected with 8 μg pcDNA3.1(-)-Hu1 or pcDNA3.1(-)-Hu2 plasmids and 2 μg pcDNA3.1(-)-TPST2 plasmid DNA, which encodes tyrosylprotein sulfotransferase 2. Trypsinogens were purified from 200 to 400 ml conditioned medium by ecotin affinity chromatography. Trypsinogens were activated with human enteropeptidase (R&D Systems), and active trypsin concentrations were determined as described later.

### Concentration determinations

Bovine trypsin was active site titrated using *p*-nitrophenyl *p*-guanidinobenzoate, as described (53). Ecotin was overexpressed in *E. coli* BL21(DE3) and purified from the periplasm using published protocols (52). The concentration of ecotin was determined by titration against the active site–titrated bovine trypsin. This ecotin batch served as an active site titrator for all human trypsins studied. Titrations were performed using trypsin concentrations (25–50 nM) at least three orders of magnitude above the equilibrium (15 h of incubation) dissociation constant (*K*_*D*_) values of ecotin, which were 8.1 and 3.2 pM for nonsulfated cationic and anionic trypsin (from *E. coli*) and 5.6 and 19.5 pM for sulfated cationic and anionic trypsins (from pancreatic juice), respectively. SPINK1 concentrations were determined by active site titration with 25 to 50 nM of the trypsin preparations used for the binding experiments. This ensured that SPINK1 and trypsin concentrations were self-consistent. The concentration of the poorly binding K41N SPINK1 variant was determined by SDS-PAGE, Coomassie Blue staining, and densitometry, using an active site–titrated SPINK1 standard, which was purified on the same day.

### Equilibrium binding assays

Binding affinity of SPINK1 to trypsins was assessed by measuring the apparent dissociation constant (*K*_*D*_) values in equilibrium, as reported previously (19, 54). Trypsin in a fixed concentration was incubated with increasing concentrations of SPINK1 inhibitor in 0.2 ml final volume in 0.1 M Tris–HCl (pH 8.0), 150 mM NaCl, 1 mM CaCl_2_, and 0.05% Tween 20 buffer for 15 h, with the exception of the K41N SPINK1 variant, which was incubated for 1 h. The free trypsin concentration in equilibrium was measured by the addition of 5 μl of 6 mM Z-Gly-Pro-Arg-AMC fluorogenic substrate, in black 96-well plates at 380 nm excitation and 460 nm emission wavelengths. Apparent *K*_*D*_ values were calculated by plotting the free trypsin concentration as a function of the total inhibitor concentration and fitting the data points to the following equation: *y* = E − (E + *x* + *K*-sqrt((E + *x* + *K*)^2^ − 4E*x*))/2, where the independent variable *x* represents the total inhibitor concentration, the dependent variable *y* is the free protease concentration in equilibrium, *K* is *K*_*D*_, and E designates the total protease concentration. Data from at least three experiments were fitted globally.

### Association rate constant measurement

The association rate of SPINK1 to trypsin was determined by a discontinuous assay, as described earlier (55). Trypsin at 50 pM was mixed with 500 pM SPINK1 in 0.1 M Tris–HCl (pH 8.0) buffer containing 150 mM NaCl, 1 mM CaCl_2_, and 0.05% Tween 20 at 23 °C. Aliquots of 150 μl were removed, and residual trypsin activity was measured by adding 3.75 μl of 6 mM Z-Gly-Pro-Arg-AMC fluorogenic substrate, in black 96-well plates at 380 nm excitation and 460 nm emission wavelengths (Fig. S1*A*). The observed pseudo–first-order rate constant, *k*_obs_, was determined from the slope of linear fits of semilogarithmic plots of ln(v_t_/v_0_) *versus* time of inhibition, where v_0_ is the maximal uninhibited enzyme activity and v_t_ is the residual trypsin activity; using the equation (–k_obs_)(time) = ln(v_t_/v_0_). The second-order association rate constant (*k*_on_) was then calculated by dividing the pseudo–first-order rate constant with the inhibitor concentration (Fig. S1*B*).

### Dissociation rate constant measurement

Protease–inhibitor complexes were prepared by incubating SPINK1 at 11 nM and trypsin at 10 nM final concentrations in 0.1 M Tris–HCl (pH 8.0) buffer containing 150 mM NaCl, 1 mM CaCl_2_, and 0.05% Tween 20 for 1 h at 23 °C. Complexes were then diluted to 50, 100, 150, and 200 pM final concentrations with the same buffer in the presence of 0.6 mM Z-Gly-Pro-Arg-AMC substrate. The increase in trypsin activity was monitored continuously for 1 h in black 96-well plates at 380 nm excitation and 460 nm emission wavelengths at room temperature (Fig. S2*A*). The parabolic curves were fitted with the second-order polynomial function *y* = a + b*x* + c*x*^2^, where the quadratic dissociation coefficient “c” equals 1/2(initial complex concentration) × (dissociation rate constant (*k*_off_)) × (turnover number). The fitted c coefficients were plotted as a function of the initial complex concentration (in pM units), and the slopes of linear fits were used to calculate *k*_off_ (in s^−1^ units) (Fig. S2*B*). The turnover number of trypsin on the peptide substrate was determined from calibration curves prepared under the same experimental conditions as used for the dissociation assay. Initial rates of substrate hydrolysis (in relative fluorescence unit/s units) were plotted against the enzyme concentrations (in pM units), and the slope of the linear fit gave the turnover number in relative fluorescence/s/pM units.

## Data availability

All data are contained within this article and the associated supporting information.

## Conflict of interest

The authors declare that they have no conflicts of interest with the contents of this article.
