# Supplementary material for: Defective binding of SPINK1 variants is an uncommon mechanism for impaired trypsin inhibition in chronic pancreatitis
Source: J Biol Chem. 2021 Jan 28;296:100343. doi: 10.1016/j.jbc.2021.100343 (PMC7949130; doi:10.1016/j.jbc.2021.100343)
Supplement: Supplementary file 1 — Figures S1 to S3 [file mmc1.pdf]

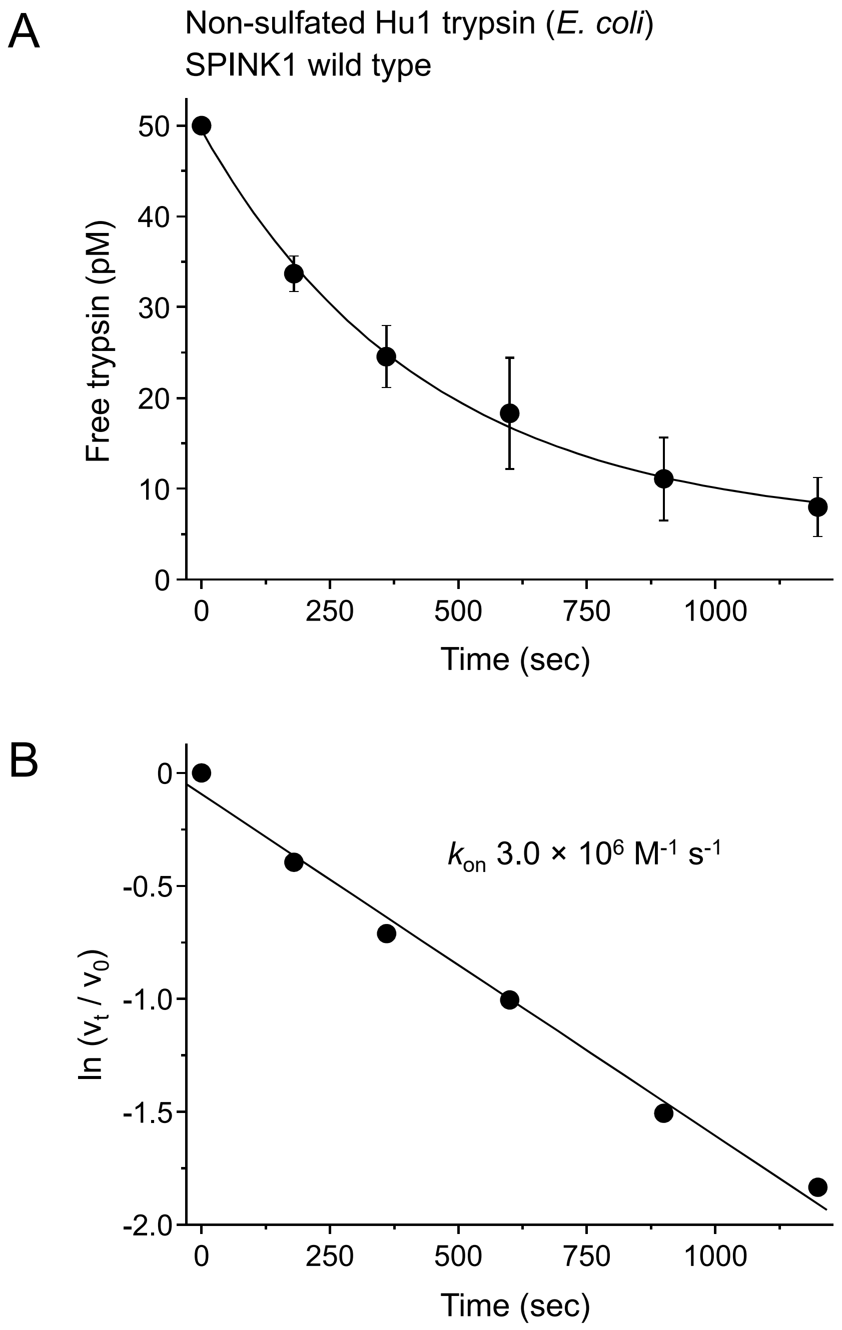

Supplementary Figure 1. Association rate constant measurement. As an example, the reaction between non-sulfated Hu1 trypsin and wild-type SPINK1 is shown. **A**, Residual trypsin activity measurement after mixing of trypsin and inhibitor. **B**, Determination of the pseudo-first order rate constant and the second-order association rate constant (k_{on}). See *Experimental Procedures* for details.

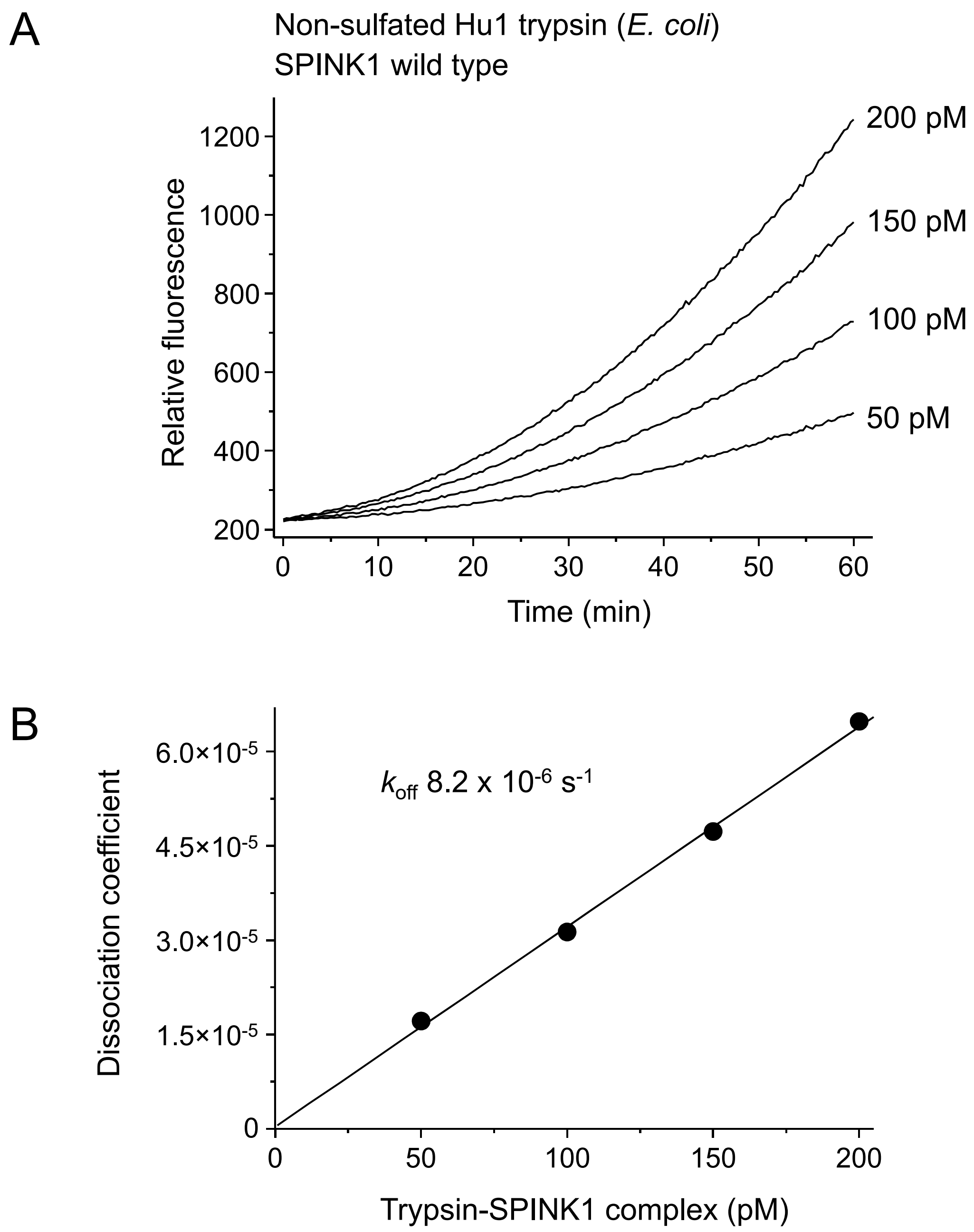

Supplementary Figure 2. Dissociation rate constant measurement. As an example, the reaction between non-sulfated Hu1 trypsin and wild-type SPINK1 is shown. **A**, Continuous measurement of trypsin activity after dilution of the trypsin-inhibitor complex. The final complex concentration after the dilution is indicated. **B**, Determination of the dissociation rate constant (k_{off}) from the quadratic dissociation coefficients. See *Experimental Procedures* for details.

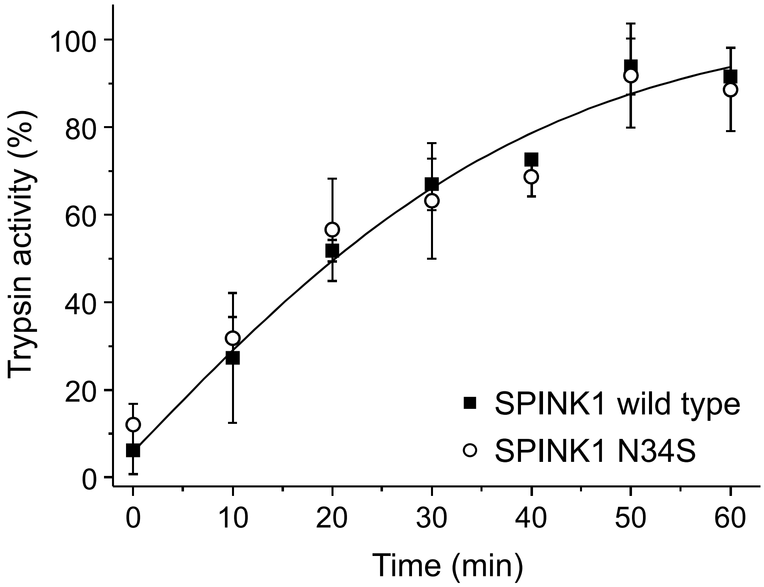

Supplementary Figure 3. Degradation of wild-type SPINK1 and the N34S variant by human mesotrypsin. SPINK1 at 0.5 μM concentration was digested with 200 nM mesotrypsin in 0.1 M Tris-HCl (pH 8.0), 1 mM $CaCl_2$ and 0.05% Tween 20 at 37 ˚C. To determine the residual SPINK1 levels, at the indicated time points 5 μL aliquots were mixed with 40 μL of 55 nM cationic trypsin in 0.1 M Tris-HCl (pH 8.0), 1 mM $CaCl_2$ and 0.05% Tween 20. After 3 min incubation at 22 ˚C, the uninhibited trypsin activity was measured by the addition of 5 μL 6 mM Suc-Ala-Ala-Pro-Lys-p-nitroanilide substrate. The trypsin activity of the 20 nM mesotrypsin carried over from the digestion mix was subtracted from the results. Loss of SPINK1 inhibitory activity was graphed as the percent gain in the activity of cationic trypsin relative to its expected full activity.
